# 143 Impact of Direct Provider Feedback on Ambulatory Antibiotic Duration for Pneumonia and Skin and Soft Tissue Infection

**DOI:** 10.1017/ash.2026.10549

**Published:** 2026-06-23

**Authors:** Lujain Malkawi, Eryiel Mascardo, Susanna Paschal, An-Lin Cheng, Ume Abbas

**Affiliations:** 1 University of Missouri Kansas City; 2 University of Missouri - Kansas City; 3 University of Missouri- Kansas City

## Abstract

**Background:** University Health (UH) Medical Center is a longstanding 238-bed safety net hospital in Kansas City, MO, that uses LabID Event Reporting for Clostridioides difficile infection (CDI), with pre-agreed intuitional testing criteria, and where there was an increase in hospital-onset (HO) CDI in 2024. We sought to examine the factors associated with HO CDI at UH. Methods We conducted a case-control study that included all patients (cases) with a positive polymerase chain reaction (PCR) for toxigenic C. difficile in 2024, on hospital day < 3. We defined HO CDI as positive PCR occurring on hospital day < 3. We matched the cases 1:1, by age and gender, with patients (controls) having a negative PCR for toxigenic C. difficile in 2024, on hospital day < 3. Electronic health record was used to extract data that included demographic and epidemiological variables, Charlson Comorbidity Index (CCI), onset of diarrhea, and timing of stool collection, length of stay, and exposures (within past six months of CDI) to hospitalization, surgery, and/or medications including laxatives, proton-pump inhibitors, immunosuppressants, and antimicrobials. Continuous variables were compared via Mann-Whitney U or t test. Categorical variables were compared using X2 or Fisher’s exact test. Univariate and multivariate logistic regression analyses were performed to assess relationship between dependent and independent variables. Statistical significance was at p< 0.05. Results In 2024, there were 20 cases of HO CDI that were matched in 1:1 ratio with 20 controls. The controls had a higher mean CCI than the cases (5.9 vs 3.5). In univariate logistic regression, eight variables had a p-value < 0.05: CCI; cephalosporin use; specialty consults; infectious disease consult; fever; ICU stay; IV contrast receipt; and liver disease. In a multivariate logistic regression model, adjusted for multicollinearity including just five variables, the CCI (OR = 0.38, p = 0.018), specialty consults (OR = 0.00, p = 0.011), and cephalosporins use (OR = 15.06, p = 0.050) were significant; In a final reduced model (removing consults), only the CCI remained significant (p = 0.023). For each one-unit increase in the index, the odds of being a case decreased by 36% (OR = 0.64). Conclusion Charlson Comorbidity Index was a negative predictor, while cephalosporin use was a positive predictor, of HO CDI at UH. Two-step C. difficile testing and tailored antimicrobial stewardship would help optimize diagnosis and management.

